# Tyrosyl-DNA phosphodiesterase 1 initiates repair of apurinic/apyrimidinic sites

**DOI:** 10.1016/j.biochi.2012.04.004

**Published:** 2012-08

**Authors:** Natalia A. Lebedeva, Nadejda I. Rechkunova, Sherif F. El-Khamisy, Olga I. Lavrik

**Affiliations:** a Institute of Chemical Biology and Fundamental Medicine, Lavrentiev av. 8, 630090 Novosibirsk, Russia; b MRC Genome Damage and Stability Centre, University of Sussex, Brighton, UK; c Department of Biochemistry, Faculty of Pharmacy, Ain Shams University, Egypt

**Keywords:** Base excision repair, AP site, Tyrosyl-DNA phosphodiesterase 1

## Abstract

Tyrosyl-DNA phosphodiesterase 1 (Tdp1) catalyzes the hydrolysis of the phosphodiester linkage between the DNA 3′ phosphate and a tyrosine residue as well as a variety of other DNA 3′ damaged termini. Recently we have shown that Tdp1 can liberate the 3′ DNA phosphate termini from apurinic/apyrimidinic (AP) sites. Here, we found that Tdp1 is more active in the cleavage of the AP sites inside bubble-DNA structure in comparison to ssDNA containing AP site. Furthermore, Tdp1 hydrolyzes AP sites opposite to bulky fluorescein adduct faster than AP sites located in dsDNA. Whilst the Tdp1 H493R (SCAN1) and H263A mutants retain the ability to bind an AP site-containing DNA, both mutants do not reveal endonuclease activity, further suggesting the specificity of the AP cleavage activity. We suggest that this Tdp1 activity can contribute to the repair of AP sites particularly in DNA structures containing ssDNA region or AP sites in the context of clustered DNA lesions.

## Highlights

► We examine an AP site cleavage activity of human Tdp1 in different DNA structures. ► Tdp1 is most active in the cleavage of AP site inside bubble-DNA structure. ► Tdp1 initiates repair of AP site opposite a bulky lesion. ► Tdp1 mutants SCAN1 and H263A fail to cleave AP site.

## 1 Introduction

Tyrosyl-DNA phosphodiesterase 1 (Tdp1) is an enzyme that catalyzes the hydrolysis of 3′-phosphotyrosyl bonds. It was shown earlier that Tdp1 shows preference to remove a tyrosine residue from the ends of single- or double-stranded DNA substrates in vitro rather than tyrosine located at a nick of duplex DNA [1–4]. It suggests involvement of the enzyme in the removal of the adducts from DNA ends rather than located at the internal sites of DNA. Human Tdp1 can also hydrolyze other 3′-end DNA alterations including phosphoglycolates and 3′-abasic sites indicating that it may function as a general 3′-DNA phosphodiesterase and repair enzyme [5]. In addition, Tdp1 possesses a 3′-nucleosidase activity in which a single nucleoside is removed from 3′-hydroxy terminated ribo- and deoxyribonucleosides [6].

Recently we have shown that Tdp1 can cleave an AP site with the formation of 3′-phosphate termini [7]. Apurinic/apyrimidinic (AP) sites arise as a result of excision of the oxidatively damaged bases by DNA glycosylases that initiate base excision repair (BER), or by spontaneous hydrolysis that generates several thousand AP sites per day in a living cell [8,9]. The major enzyme in eukaryotic cells that catalyzes the cleavage of AP sites is apurinic/apyrimidinic endonuclease 1 (APE1). APE1 hydrolyzes the phosphodiester bond on the 5′-side of the abasic sites. We found that human Tdp1 also can initiate repair of AP sites. The 3′-phosphates generated by Tdp1 are efficiently removed by polynucleotide kinase 3′-phosphatase (PNKP) to produce a 3′-hydroxyl, which can be processed further and repaired by DNA polymerases and ligases [10–12]. Tdp1 is known to interact with base excision repair proteins: DNA polymerase beta (Pol β), XRCC1, poly(ADPribose)polymerase 1 (PARP1) and DNA ligase III [5,13–15]. A role for Tdp1 to maintain mitochondrial genetic integrity has recently been proposed [16] and a role in clearing alkylation DNA damage *in vivo* has also been reported for yeast *Schizosaccharomyces pombe* [17].

The reaction catalyzed by Tdp1 involves a covalent intermediate in which an active site histidine (His263 in human Tdp1) is linked by a phosphamide bond to the DNA 3′-phosphate of the substrate [18,19]. The importance of Tdp1 in humans is highlighted by the observation that a recessive mutation in the *TDP1* is responsible for the inherited disorder, spinocerebellar ataxia with axonal neuropathy (SCAN1) [15,20] in which a H493R mutation in the Tdp1 causes the accumulation of both Top1-DNA and Tdp1-DNA covalent intermediates *in vivo* [13,21–23].

In the present study we analyzed the AP site cleavage activity of Tdp1 using different DNA substrates and examined the possibility to repair AP sites located opposite to bulky DNA lesions. Our data suggest a novel APE-independent pathway for processing AP sites.

## 2 Materials and methods

### 2.1 Materials

[γ^32^P]ATP (5000 Ci/mmol) was produced in the Laboratory of Radiochemistry (ICBFM, Novosibirsk); phage T4 polynucleotide kinase was purchased from Biosan (Russia); stained molecular mass markers were from Fermentas (Lithuania), reagents for electrophoresis and buffer components from Sigma (USA). Ultrapure dNTPs were from SibEnzyme (Russia).

The recombinant wild-type Tdp1 and mutant human Tdp1 proteins (SCAN1 and H263A) were purified to homogeneity by the chromatography on Ni-chelating resin and phosphocellulose P11 as described [18]. Wild-type Tdp1 was additionally purified by gel filtration through S-200 Sephacryl column using buffer containing Triton X-100 (0.01%) and 0.15 M NaCl followed by chromatography on heparin sepharose. The recombinant plasmid coding mutant Tdp1 (H263A) was a generous gift from Dr. James Champoux (University of Washington, Seattle). The recombinant purified UDG and DNA polymerase β were a generous gift from Dr. S.N. Khodyreva (ICBFM, Novosibirsk). The recombinant purified XRCC1 was a generous gift from Dr. I.A. Vasil'eva (ICBFM, Novosibirsk). PNKP and DNA ligase III were kindly donated by Dr. D.O. Zharkov (ICBFM, Novosibirsk).

### 2.2 Radioactive labeling of oligonucleotides

**Fig. 1:**
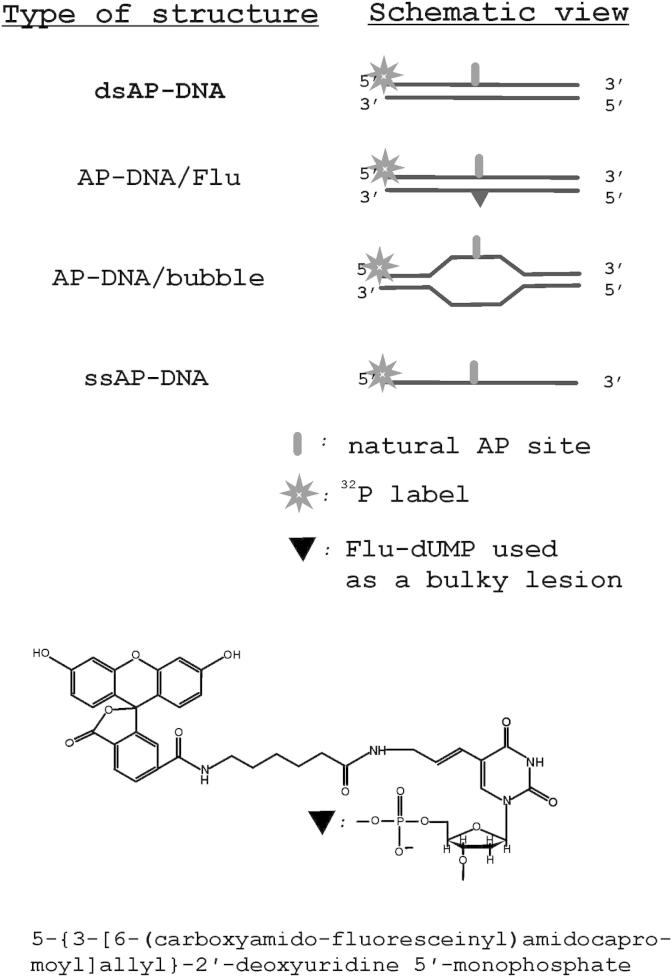
Schematic view of DNA structures and nucleotide analog used in the experiments.

Oligodeoxynucleotides were 5′-[^32^P]-labeled with T4 polynucleotide kinase and [γ^32^P] ATP as described [24]. Unreacted [γ^32^P] ATP was removed by passing the mixture over a MicroSpin™ G-25 column (Amersham, USA) using the manufacturer's suggested protocol. Complementary oligodeoxynucleotides were annealed in equimolar amounts by heating a solution to 95 °C for 3 min, followed by slow cooling to room temperature. The sequences of the oligonucleotides used in experiments were as follows:

ssAP-DNA
5′-ctat ggcg aggc gatt aagt tggg **U**ac gtca gggt cttc cgaa cgac
dsAP-DNA
5′-ctat ggcg aggc gatt aagt tggg **U**ac gtca gggt cttc cgaa cgac
3′-gata ccgc tccg ctaa ttca accc gttg cagt ccca gaag gctt gctg
AP-DNA/Flu
5′-ctat ggcg aggc gatt aagt tggg **U**ac gtca gggt cttc cgaa cgac
3′-gata ccgc tccg ctaa ttca accc g**F**tg cagt ccca gaag gctt gctg
AP-DNA/bubble
5′-ctat ggcg aggc gatt aagt tggg **U**ac gtca gggt cttc cgaa cgac
3′-gata ccgс tccg ctaa tagt tggg taac gtca ccca gaag gctt gctg,

where U designates dUMP residue, which is converted to AP site by the following UDG treatment; F designates 5-{3-[6-(carboxyamidofluoresceinyl)amidocapromoyl]allyl}-2′-deoxyuridine-5′-monophosphate residue (Flu-dUMP, see Fig. 1 for structure).

### 2.3 Endonuclease assays

Standard reaction mixtures (10 μl) contained 50 mM Tris–HCl (pH 7.5), 50 mM NaCl, 5 mM MgCl_2_, 10 nM 5′-[^32^P]-labeled DNA substrate and necessary enzymes (Tdp1, SCAN1 or H263A). For the preparation of natural AP site, an AP-DNA duplex was first incubated in reaction buffer with UDG (0.5 U/μl) for 15 min at 37 °C. After adding Tdp1 (100 nM) the reaction mixtures were incubated at 37 °C for 30 min. Then reactions were terminated by adding of the formamide dye and the mixtures were heated for 3 min at 90 °C. The products were analyzed by electrophoresis in 20% polyacrylamide gel with 8 M urea followed by autoradiography [24].

### 2.4 DNA repair reconstitution assay

The reaction mixture (10 μl) contained 10 nM of the labeled substrate in a buffer containing 50 mM Tris–HCl (pH 7.5), 50 mM NaCl, 5 mM MgCl_2_, 0.5 mM dCTP, 1 mM ATP. Different combinations of Tdp1 (100 nM), Pol β (50 nM), XRCC1 (10 nM), PNKP (300 nM), and DNA ligase III (10 nM) or T4 DNA ligase (200 U/μl if indicated) were added as required. The mixtures were incubated at 37 °C for 30 min and analyzed as above.

### 2.5 Protein binding to DNA

Protein-DNA complexes were analyzed by gel retardation. The reaction mixture (10 μl) contained 50 mM Tris–HCl, pH 7.5, 50 mM KCl, 10 nM 5′-^32^P-labeled DNA, and wild-type Tdp1 or mutants of Tdp1 at various concentrations. AP-DNA duplex was first incubated in reaction buffer with UDG (0.5 U/μl) for 15 min at 37 °C. The complexes Tdp1 with DNA were formed on ice. Then loading buffer (1:5 v/v) containing 20% glycerol and 0.015% Bromophenol Blue was added to the sample. Protein–nucleic acid complexes were electrophoresed under nondenaturing conditions. To separate the products of complex formation of Tdp1 or mutants, 5% polyacrylamide gel (acrylamide/bis-acrylamide = 60:1) was used. TBE was the electrode buffer. Electrophoresis was performed with voltage decrease 17 V/cm and at 4 °C. Positions of radioactively labeled oligonucleotide and protein–nucleic acid complexes were determined autoradiographically using a Molecular Imager FX Pro+ from BioRad.

## 3 Results and discussion

### 3.1 AP site cleavage activity of Tdp1 depends on DNA structure

**Fig. 2:**
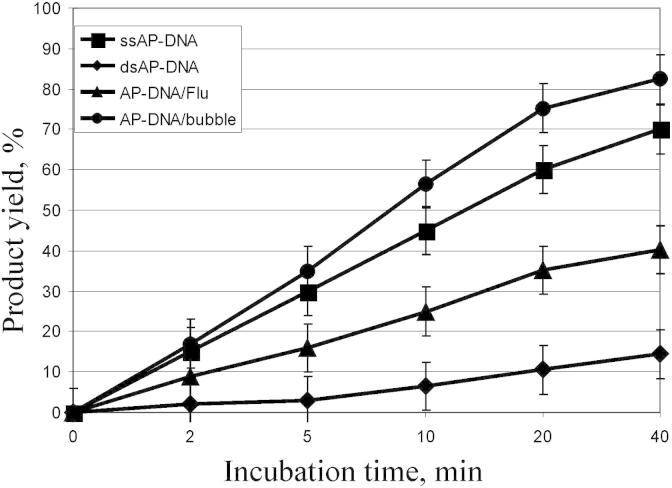
Kinetics of Tdp1 cleavage activity on different AP-DNA substrates. The AP-DNA substrates were treated from 1 to 40 min with 100 nM Tdp1 after incubation DNA with UDG (0.5 U/μl) for 15 min at 37 °C. The percent conversion from the substrate to repair products was calculated. The experiment was performed in triplicate, and the standard deviation for each point is indicated by error bars. Closed circles, AP-DNA/bubble; closed squares, ssAP-DNA; closed triangles, AP-DNA/Flu; closed rhombus, dsAP-DNA.

Recently we reported that human tyrosyl-DNA phosphodiesterase 1 (Tdp1) catalyzes the apurinic/apyrimidinic site (AP site) cleavage reaction to generate breaks with the 3′- and 5′-phosphate termini [7]. To further investigate this activity we analyzed the capability of Tdp1 to hydrolyze AP sites in different DNA structures. The ssDNA, dsDNA, DNA structure with bubble and AP sites located opposite the bulky lesions in dsDNA were used as substrates for Tdp1 (Fig. 1). The kinetics of cleavage of AP sites in these structures are shown in Fig. 2. In the previous study we reported that Tdp1 cleaved AP site in ssDNA more effectively than in duplex DNA structure [7]. Here we demonstrate that Tdp1 is more active in the cleavage of AP site inside bubble-DNA structure in comparison with ssDNA containing AP site. AP site located opposite to bulky fluorescein adduct in the other strand of DNA duplex is hydrolyzed by Tdp1 faster than in dsDNA containing AP site (Fig. 2). One can see that the efficiency of AP site hydrolysis catalyzed by Tdp1 decreases in the order: dsAP-DNA/bubble > ssAP-DNA > dsAP-DNA/Flu >> dsAP-DNA.

### 3.2 Tdp1 activity is affected by the position of AP sites within DNA

**Fig. 3:**
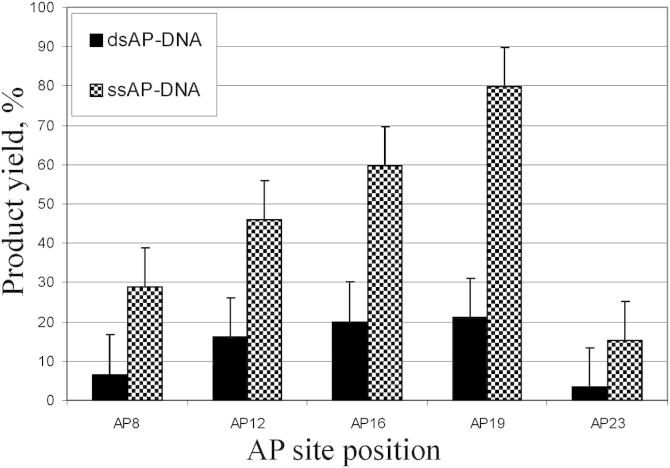
The influence of AP site position in DNA structures on the Tdp1 activity. Graph of Tdp1 activity measured as the percentage of AP-DNA substrates cleaved to product. Defined amounts of Tdp1 (100 nM) were incubated for 30 min with 10 nM ssDNA substrate or dsDNA duplex containing AP site.

Since Tdp1 showed preference for 3′-substituent located on the termini of DNA compared to nicks [2,3], we decided to examine an influence of AP site position in DNA structures on the Tdp1 cleavage efficiency. We quantified Tdp1 activity on 32-mer ssDNA and DNA duplexes bearing AP site at positions 8, 12, 16, 19, and 23 from the 5′ end (Fig. 3). The efficiency of AP site hydrolysis increased when AP site was shifted from the 5′ end in both ssDNA and DNA duplex. The highest efficiency was observed with AP site at position 19. Surprisingly, the cleavage efficiency was dramatically reduced at position 23. It should be noted that AP sites at positions 3 and 30 as well as AP sites within DNA substrates of 12 and 17 nucleotides were resistant to Tdp1 (data not shown). Recently Interthal and Champoux reported that the affinity of Tdp1 for the DNA substrate increases as the length of the DNA is increased from 6 to 19 nucleotides and modestly increases further upon the extension of the length to 28 nucleotides [25]. To explain these observations the authors speculate that Tdp1 may initially bind to DNA at internal sites before the 3′ end is inserted in the active center of the enzyme. This suggestion is in line with our observation that Tdp1 preferably cleaves AP sites located in the middle of the strand but does not explain the fact that AP sites located near to the 3′ end are refractory to cleavage by Tdp1, whereas enzyme retains the ability to remove the 3′ terminal nucleoside in all tested structures.

### 3.3 Repair of AP sites in cluster with bulky DNA lesions initiated by Tdp1

**Fig. 4:**
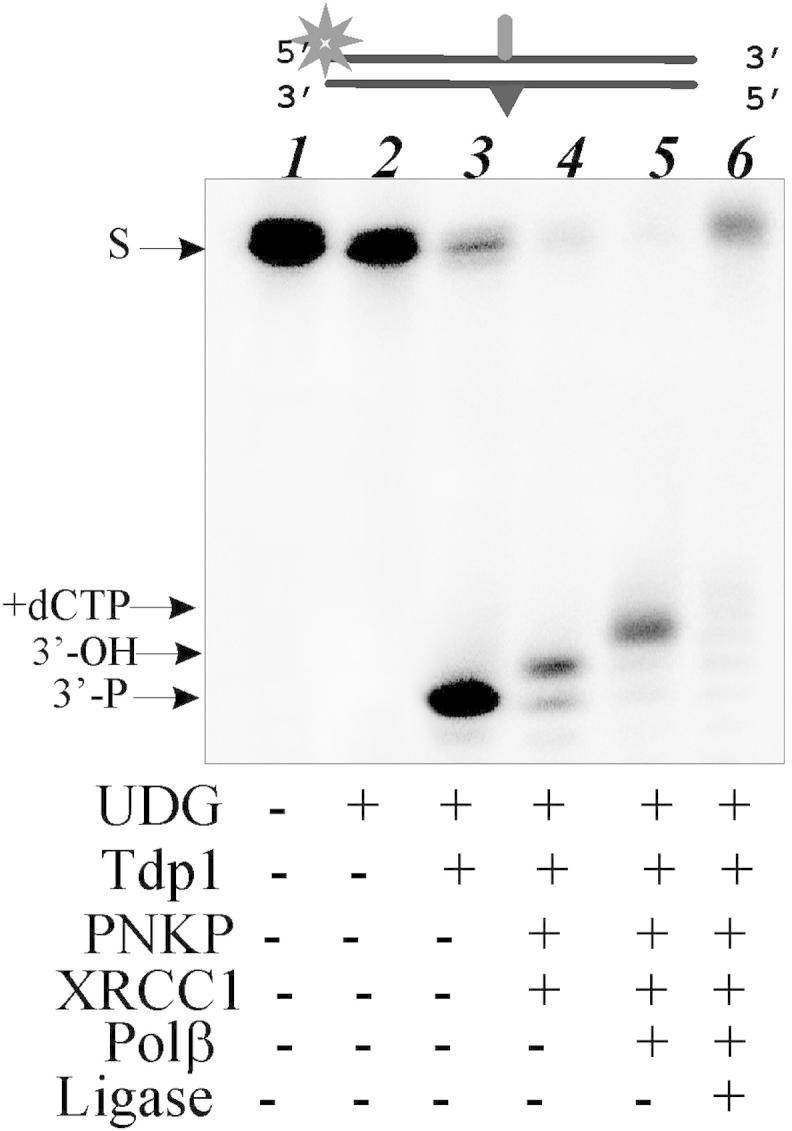
Repair of AP site initiated by Tdp1 in DNA duplexes containing AP site in cluster with bulky lesion. The 5′-end labeled AP-DNA substrate was subsequently incubated with the UDG (lane 2), Tdp1 (lane 3), PNKP and XRCC1 (lane 4), Pol β (lane 5), DNA ligase III (lane 6) to monitor DNA repair. Lane 1 corresponds to the AP-DNA. The components present in different reaction mixtures are indicated.

Due to the more efficient cleavage of AP sites catalyzed by Tdp1 in the case of ssDNA compared to dsDNA, we wondered whether this activity may be important to initiate repair of AP sites in the context of cluster-type lesions when AP sites located nearby bulky lesions. AP sites could be components of clustered DNA lesions located within one or two turns of DNA. Cluster lesions result from intensive oxidative stress, X-ray or UV light irradiation or environmental mutagenic action on DNA. Repair of cluster DNA lesions is one of the most difficult tasks in eukaryotic cells. Therefore, we analyzed the role of Tdp1 during the repair of AP sites in cluster with bulky lesion using reconstituted BER system consisting of purified proteins (Fig. 4). A 5′ ^32^P-labeled 48-mer DNA duplex containing uridine in one strand opposite to fluorescein on the other strand (Fig. 1, AP-DNA/Flu) was incubated with purified recombinant UDG, Tdp1, PNKP, Pol β, DNA ligase III, and XRCC1. The reaction mixture containing Tdp1 but lacking PNKP generated a product with a 3′-phosphate, which is identical to that produced by NEIL1 (Fig. 4, lane 3). Addition of PNKP resulted in a product with the 3′-OH termini (Fig. 4, lane 4). Lastly, DNA polymerase β replaces the missing DNA segment (Fig. 4, lane 5) and DNA ligase reseals the DNA (Fig. 4, lane 6). So, the repair of AP sites initiated by Tdp1 fully restored the intact DNA and generated the products of the expected lengths at each intermediate stage. In summary, human Tdp1 can initiate APE1-independent repair of AP sites that expands the ability of the BER process.

It is interesting that Tdp1 can function in a fashion similar to NEIL1, bifunctional DNA glycosylase involved in BER, because both enzymes produce 3′-phosphate after the cleavage of AP site. The following processing of this DNA intermediate is also dependent on PNKP. However NEIL1 was unable to cleave DNA with tetrahydrofuran moiety whereas TDP1 efficiently cleaved this structure [7]. This feature allows us to suggest different mechanisms possessed by NEIL1 and Tdp1 during the repair of AP sites. The significance and details of the Tdp1 mechanism should be analyzed further.

### 3.4 Tdp1 mutants H493R (SCAN1) and H263A fail to hydrolyze AP site

**Fig. 5:**
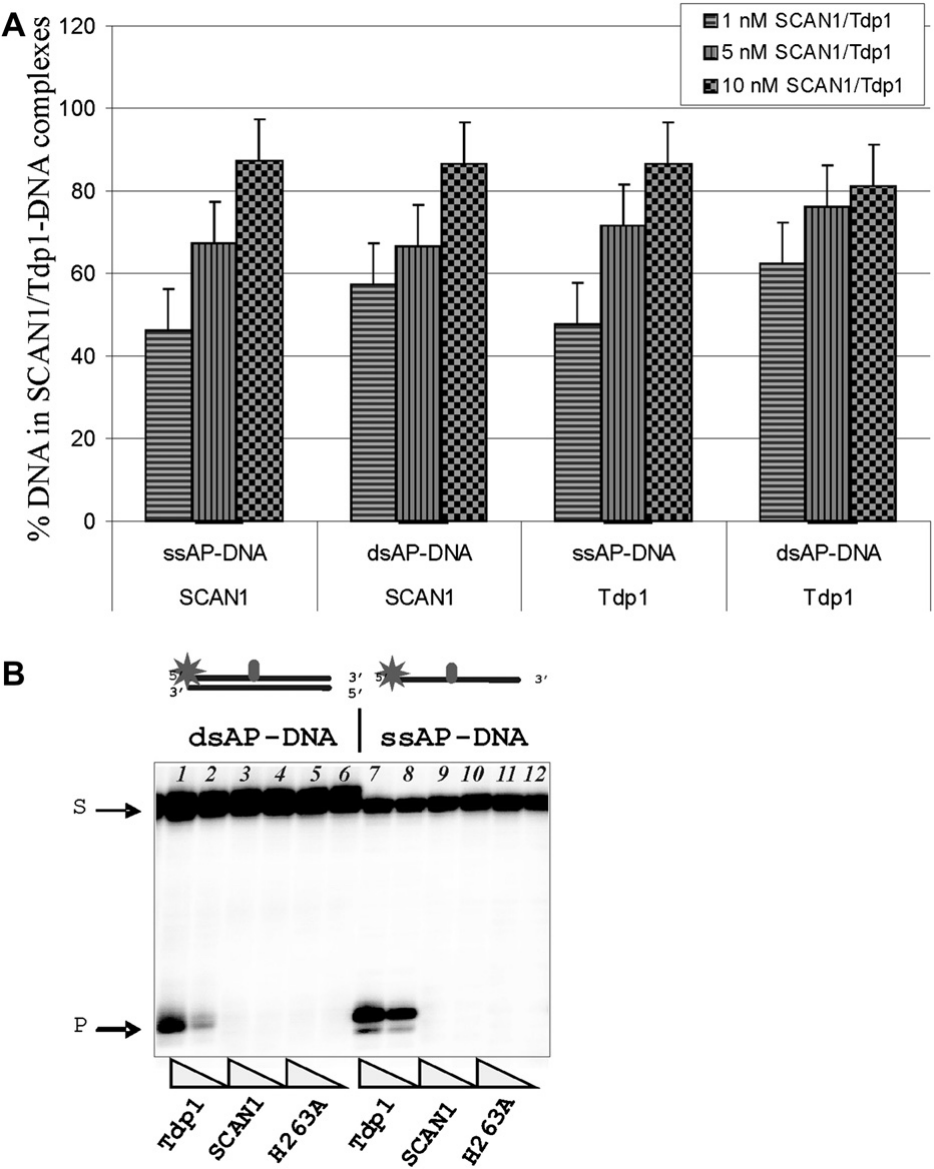
Binding and AP site cleavage by Tdp1 and its mutants. The A panel shows quantitative analysis of the data from three independent of gel shift experiments. The 5′-end labeled DNA substrates (ssAP-DNA or dsAP-DNA) were incubated with increasing concentrations of Tdp1 or its mutants (from 1 nM up to 10 nM) and subjected to native gel electrophoresis. The gels were dried and analyzed using the PhosphorImager. The **B** panel shows the hydrolysis of AP sites in single (lane 7–12) and double (lane 1–6) stranded DNAs by Tdp1 and its mutants. The 5′-end labeled AP-DNA was incubated with decreasing concentrations of Tdp1 (lanes 1, 2, 7, 8), SCAN1 (lanes 3, 4, 9, 10), H263A (lanes 5, 6, 11, 12). S denotes the initial substrate, the 48-mer DNA duplex.

The catalytic mechanism proposed for Tdp1 on the basis of protein structure includes two histidine residues, namely His263 and His493, in the active center [18,19]. Here we utilized two mutants of Tdp1 in which one of these histidines have been replaced. The H493R mutation, when the histidine 493 is replaced by arginine, is responsible for the autosomal recessive neurodegenerative disease, spinocerebellar ataxia with axonal neuropathy (SCAN1). In the second mutant, the histidine 263 is replaced by alanine (H263A). To analyze an involvement of these residues in the AP site hydrolysis we have investigated binding to AP-DNA and activity in the reaction of AP site cleavage of these Tdp1 mutants. DNA binding experiments indicate that both mutants retain the ability to bind AP site-containing DNA with similar affinity as wild-type Tdp1 (Fig. 5A). In striking contrast, using ssDNA and dsDNA structure containing AP sites we show that both mutants were catalytically inactive with regard to processing AP sites (Fig. 5B).

Taken together, our results suggest that the AP site cleavage activity of human Tdp1 may play an important role in repair of AP sites, particularly in the context of cluster-type lesions when AP sites are in close proximity to bulky adducts in DNA. Therefore, this new activity of tyrosyl-DNA phosphodiesterase 1 can contribute to the repair of AP sites and this pathway is independent on the AP endonuclease 1 activity. Whether this activity is influenced by Tdp1 posttranslational modifications, such as phosphorylation [14,26] or SUMOylation [27] and its significance for Tdp1 cellular function is yet to be determined.

## Disclosure statement

The authors declare that they have no competing interests.

## Authors' contributions

NL performed most of the experiments and wrote the initial draft. NR purified the recombinant proteins and edited the manuscript. SEK provided plasmids and discussed the results. OL designed, coordinated the study, and edited the manuscript. All authors read and approved the final manuscript.
